# Nano Carbon Doped Polyacrylamide Gel Electrolytes for High Performance Supercapacitors

**DOI:** 10.3390/molecules26092631

**Published:** 2021-04-30

**Authors:** Samar Azizighannad, Zhiqian Wang, Zain Siddiqui, Vivek Kumar, Somenath Mitra

**Affiliations:** 1 Department of Physics and Materials Science, New Jersey Institute of Technology, Newark, NJ 07102, USA; sa698@njit.edu; 2 Department of Chemistry and Environmental Science, New Jersey Institute of Technology, Newark, NJ 07102, USA; zhiqian.wang@njit.edu; 3 Department of Biomedical Engineering, New Jersey Institute of Technology, Newark, NJ 07102, USA; zs67@njit.edu (Z.S.); vak@njit.edu (V.K.)

**Keywords:** polymer electrolyte, gel electrolyte, supercapacitor, graphene oxide, carbon nanotube

## Abstract

Novel polyacrylamide gel electrolytes (PGEs) doped with nano carbons with enhanced electrochemical, thermal, and mechanical properties are presented. Carboxylated carbon nanotubes (fCNTs), graphene oxide sheets (GO), and the hybrid of fCNT/GO were embedded in the PGEs to serve as supercapacitor (SC) electrolytes. Thermal stability of the unmodified PGE increased with the addition of the nano carbons which led to lower capacitance degradation and longer cycling life of the SCs. The fCNT/GO-PGE showed the best thermal stability, which was 50% higher than original PGE. Viscoelastic properties of PGEs were also improved with the incorporation of GO and fCNT/GO. Oxygen-containing functional groups in GO and fCNT/GO hydrogen bonded with the polymer chains and improved the elasticity of PGEs. The fCNT-PGE demonstrated a slightly lower viscous strain uninform distribution of CNTs in the polymer matrix and the defects formed within. Furthermore, ion diffusion between GO layers was enhanced in fCNT/GO-PGE because fCNT decreased the aggregation of GO sheets and improved the ion channels, increasing the gel ionic conductivity from 41 to 132 mS cm^−1^. Finally, MnO_2_-based supercapacitors using PGE, fCNT-PGE, GO-PGE, and fCNT/GO-PGE electrolytes were fabricated with the electrode-specific capacitance measured to be 39.5, 65.5, 77.6, and 83.3 F·g^−1^, respectively. This research demonstrates the effectiveness of nano carbons as dopants in polymer gel electrolytes for property enhancements.

## 1. Introduction

The development of new generation portable and flexible electronics has increased the demand for lightweight, flexible, long cycle life, high performance, and safe energy storage devices [1,2,3,4]. Supercapacitors (SC) are essential energy storage devices [5,6] that play a crucial role in increasing battery life and energy efficiency [7,8,9,10]. Additionally, SCs are candidates to replace rechargeable batteries due to their high-speed charge-discharge capabilities, high power densities, low costs, and long cycle life [11]. Along with enhancing electrode capacitance, there has been much interest in improving the electrochemical potential of electrolytes [12,13].

SC electrolytes can be liquid, gel, and solid [14]. Liquid electrolytes have low dynamic viscosity and high conductivity compared to gel and solid electrolytes [15]. However, their shortcomings are safety, high costs of packaging, narrower operating temperature windows, and lower decomposition voltages [16,17,18,19,20]. It is also difficult to package in flexible and conformal devices. Flexible electrodes along with solid-state electrolytes have been used in flexible devices [21]. While they have good mechanical properties and are very suited to flexible devices, the solid electrolytes typically show low ionic conductivity and overall performance. Gel polymer electrolytes (GE) are a great compromise between the liquid and solid, have relatively good mechanical properties, are lightweight, can be packaged easily, and can have higher ionic conductivity than the solids [22,23,24,25,26].

Designing composites with optimal thermal, mechanical, and electrochemical properties is an important consideration in the new generation GE development [27,28,29,30,31,32,33,34,35]. A variety of aqueous, non-aqueous, redox and ionic liquid GEs has been reported. [17,27]. Polymers such as poly(acrylonitrile) (PAN) [28], poly(vinyl alcohol) (PVA) [29], polyaniline (PANI) [30], poly(ethylene oxide) (PEO) [31], poly(methyl methacrylate) (PMMA) [32], and poly(vinylidene fluoride) (PVDF) [33] have been studied for gel polymer electrolyte applications. Another important parameter in GE synthesis is the electrolyte selection such as acidic, alkali and neutral. PVA is the most commonly used GE with KOH electrolyte but has several limitations such as low ionic conductivity and mechanical strength, and limited work has been reported for neutral electrolytes such as lithium chloride (LiCl) [27,34]. At this point, there is a need to explore other gel electrolytes, especially for neutral electrolytes such as Li^+^ which have lower corrosion and lower water content. Polyacrylamide (PAM) is a promising candidate for gel electrolytes with higher ionic conductivity due to its porous nature and improved mechanical properties [36]. PAM along with lithium sulphate (LiSO_4_) is potentially a good combination for fabricating neutral PGE as Li with its small radius can show faster diffusion.

Typical GEs suffer from low ionic migration compared to liquid electrolytes and it is crucial to design a new generation of GEs with proper ionic conductivity, mechanical strength and thermal stability for better performance SCs. Carbon-based nanomaterials have excellent thermal, electrical, and mechanical properties and are among the most commonly used additives to improve electrolytes as well as electrodes [13,29,37]. The incorporation of carboxyl functionalized carbon nanotubes (fCNTs) and graphene oxide (GO) into PGEs have been shown to alter ionic conductivity and mechanical properties in PVA aqueous alkali, PAM non-aqueous and PVDF ionic liquid gel electrolytes [34,38,39]. Moreover, using carbon-nanomaterials in electrolytes also can increase electrode/electrolyte compatibility. The objective of this study was to develop novel neutral PAM-based GEs incorporated with nanocarbons in order to improve thermal, mechanical and electrochemical properties.

## 2. Results and Discussion

### 2.1. Morphology of PGEs

**Figure 1:**
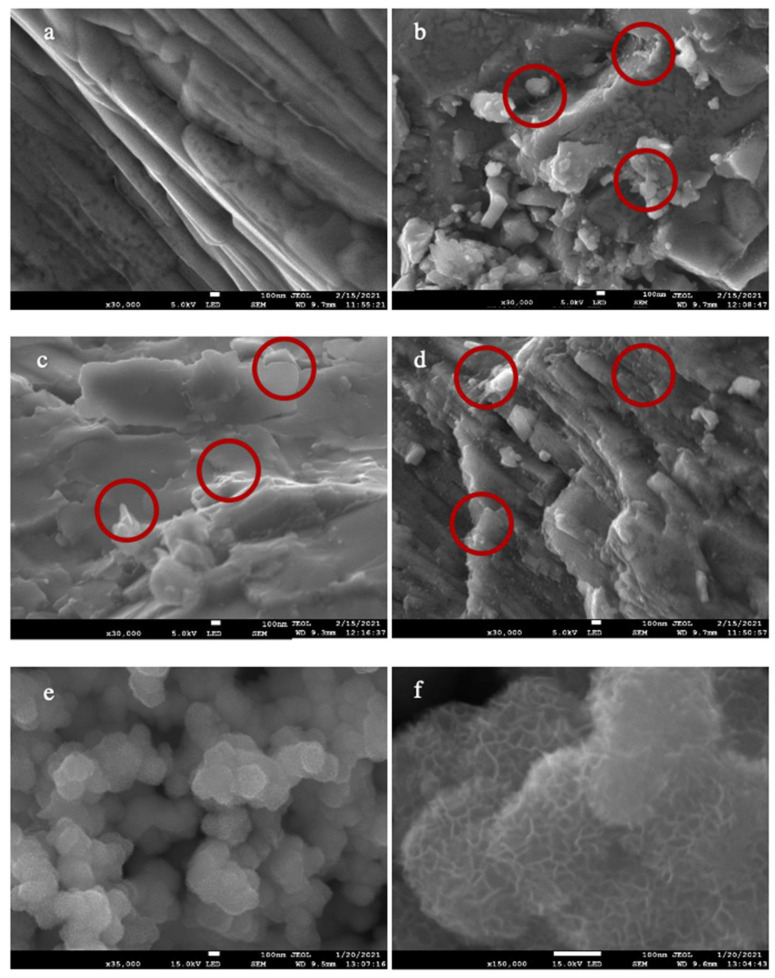
SEM images of (**a**) PGE, (**b**) fCNT-PGE, (**c**) GO-PGE, (**d**) fCNT/GO-PGE, (**e**) and (**f**) Generated MnO_2_ for electrode materials.

**Figure 2:**
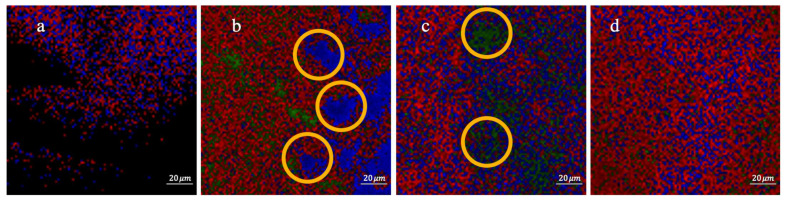
MCR images of (**a**) PGE, (**b**) fCNT-PGE (Blue color corresponds to fCNTs), (**c**) GO-PGE (Green color corresponds to GO), (**d**) fCNT/GO-PGE.

Scanning Electron Microscope (SEM) images of PGEs and electrode material are presented in Figure 1 and Figure 2. Figure 1a shows a flat structure of the dried PGE without any additive. Figure 1b–d show the structures of the doped GEs by nano carbons, namely fCNTs, GO, fCNT/GO in the gel structures. fCNTs tended to tangle and cluster, while the GO layers were stacked and agglomerated, causing the uneven distribution of nanomaterials through the PGEs. On the other hand, in the fCNT/GO hybrid, there are π-π stacking interactions between fCNTs and graphene layers, which increase synergistic effects in the hybrids [40] in the gel scaffolds [41]. Figure 1e,f show synthesized MnO_2_ as active material in SCs. Roughness and wrinkles on the synthesized MnO_2_ increased the surface area and improved the overall performance of SCs.

To further study the distribution of carbon nanomaterials in the PGE structure, Raman chemical imaging was performed. A 523 nm excitation laser was selected for an exposure time of 0.05 s in five scans. Multivariate Curve Resolution (MCR) was used to investigate the composition via color coding with 50x magnification [42] and the images of PGEs are presented in Figure 2. Each color on the MCR image represents a component in the PGE, and each component’s identification was based on the Raman spectrum. Figure 2a shows the uniform structure of the PGE matrix, and the black color shows saturation of the individual Raman spectrum. Figure 2b,c show an accumulation of fCNTs (blue) and GO (green) in the gel structure, respectively. From chemical composition images, it is observed that the distribution of the doping materials throughout the fCNT/GO-PGE was uniform, and all the components diffused evenly (Figure 2d).

### 2.2. Thermal Stability Analysis

The thermal stability of the GE plays an important role in SC stability and its life cycle. Heat is produced and temperature rises during the SC charge-discharge process, followed by electrolyte decomposition and device failure if not properly treated. These even lead to safety issues. Additionally, the performance of SCs is expected to remain stable in high-temperature environments [16,43].

**Figure 3:**
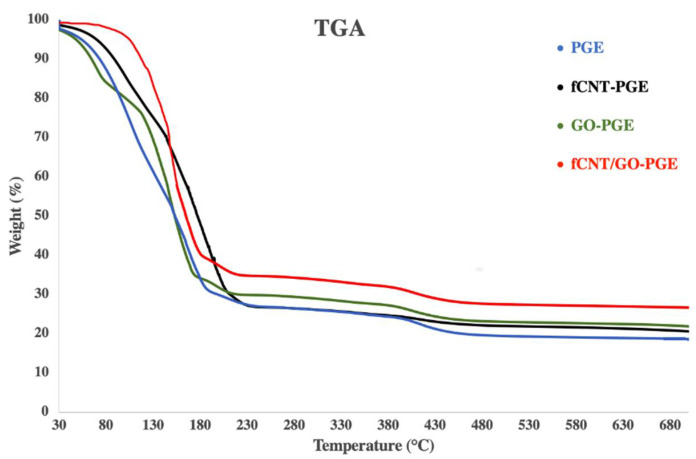
TGA graphs of PGE, fCNT-PGE, GO-PGE and fCNT/GO-PGE.

The thermal stability of GEs was evaluated by a Thermo Gravimetric Analyzer (TGA) and is presented in Figure 3. Measurements were performed under heating the temperature from 30 to 700 °C at a heating rate of 10 °C/min. Thermal degradation occurred in three steps. The first weight loss was from removal of moisture. During the next stage, the co-polymer degraded (from 150 to 430 °C). The addition of GO and fCNTs enhanced the thermal stability. Since GO sheets had higher oxygen contents (~45%), the weight loss at the initial stage was more significant than the others. fCNT/GO-PGE showed the best thermal stability among tested samples which would cause the lowest capacitance degradation.

Differential Scanning Calorimeter (DSC) measurements were performed from 30 to 300 °C in the presence of N_2_ gas at a heating rate of 10 °C/min and cooling from 300 to 30 °C at the same rate of 10 °C/min. DSC is a useful for measuring the thermodynamic properties of the gel electrolytes. Glass transition temperature (T_g_) and the melting enthalpy (ΔH_m_) based on DSC curves are presented in Table 1. According to Table 1, T_g_ of the electrolytes increased upon adding NCs due to strong intermolecular interaction between the NCs and the polymer chains. This interaction also reduced chain mobility. Since GO contained more oxygen-containing functional groups than fCNTs, the hydrogen bonds within GO-PGE were more abundant than in fCNT-PGE. More effective hydrogen bonds in the electrolyte structures brought a higher T_g_ for fCNT/GO-PGE compared to other electrolytes. The gel is a mixture containing water, which is removed between 150 and 200° C. After that, only the polymer is left. The melting enthalpy is that of the polymer composite rather than the gel itself. Reduction in ∆H_m_ from 177 to 91 J/g indicated decreasing crystallinity of GEs after doping with carbon nanomaterials. Lower alignments in polymer chains also formed canals within the gels for better ion diffusion.

### 2.3. Viscoelastic Properties of GEs

**Figure 4:**
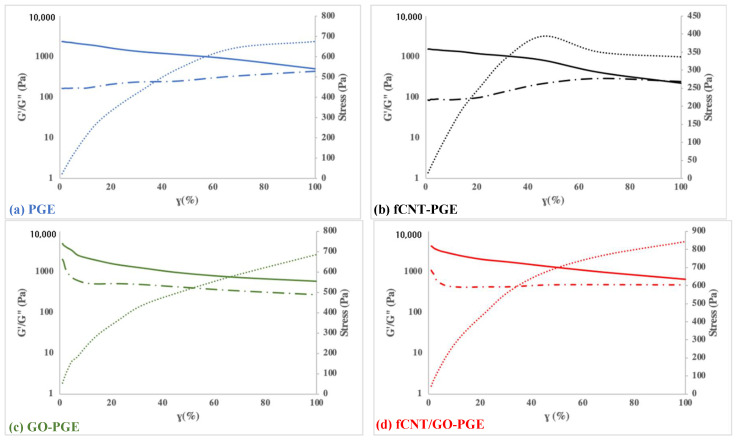
Strain sweep and strain stress curves of (**a**) PGE, (**b**) fCNT-PGE, (**c**) GO-PGE, (**d**) fCNT/GO-PGE.

The rheological behaviors of samples were studied with an oscillator. A dynamic strain sweep test was performed under the constant strain amplitude of 1 Hz at room temperature to investigate the effect of shear stress on the viscoelastic behavior of the GEs. Storage (elastic) modulus (G′) and the loss (viscous) modulus (G″) were collected and presented as a function of strain percentage in Figure 4. The increment of G′ in GO-PGE and fCNT/GO-PGE highlights the viscoelastic properties of the viscous gel into a stiffer and elastic composite. This alteration may be due to that the oxygen-containing groups present in GO and fCNT/GO group formed hydrogen bonds with polymer chains, potentially improving the mechanical strength of the gel [34].

Conversely, fCNTs that contained fewer carboxyl groups than GO agglomerated more within the PGE complex, forming defect areas. Nanomaterial agglomeration also caused a lack of hydrogen bonds between the fCNTs and polymer matrix. The ratio of G″ to G′ introduced as loss factor (tan δ) is presented in Table 1. Tan δ describes the s elastic (G′) and viscous (G″) characteristics of the PGEs, specifically when the ratio of G″ to G′ is equivalent to 1. The fCNT-PGE in Figure 4b highlighted this interaction: when the externally applied force was greater than intermolecular forces of the material, an irreversible collapse would occur [44]. A lower loss factor for PGE and fCNT-PGE represented a more solid-like structure, while a higher loss factor for GO-PGE and fCNT/PGE represented a more gel-like sticky structure. Adhesive properties are desired properties in SC applications in order to increase the contact between electrolytes and electrodes [45].

### 2.4. EIS and CV Analysis for Gel Electrolytes

**Figure 5:**
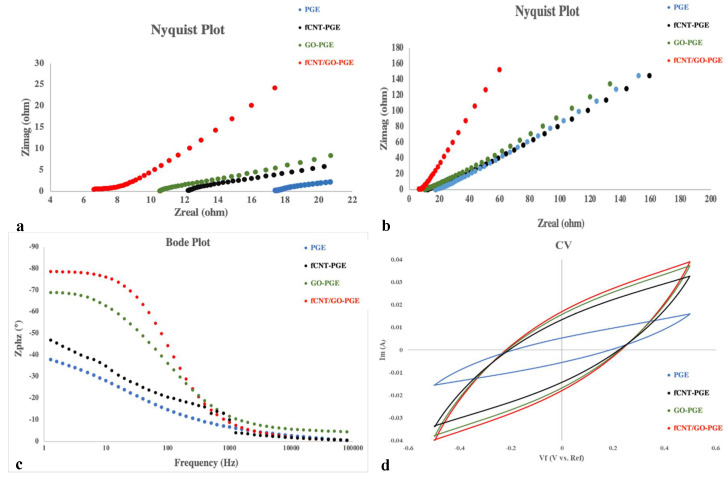
(**a**,**b**) Nyquist plots, (**c**) Bode plots, and (**d**) Cyclic voltammetry at 100 mV s^−1^ of PGE, fCNT-PGE, GO-PGE, fCNT/GO-PGE.

Electrochemical impedance spectroscopy (EIS), an alternating current (AC) based measurement was performed to reach detailed information about PGEs [46]. The efficiency of SCs strongly depends on ion redox and ionic conductivity of the electrolytes. Two probe analysis was carried out by connecting a working/sense electrode to an anode graphite sheet and counter/reference electrode to a cathode graphite. The electrode graphite sheets with controlled areas were placed in a three-dimensional printed case filled with gel electrolytes. After the formation of the GPEs, probes were connected to the graphite sheets. The distance between the electrodes was also controlled based on the casing designs. Measurements were performed under 10 mHz to 100 kHz with 5 mV amplitude and 0 DC voltage, and an oscillating current response through the sample was collected. Nyquist and bode plots presented in Figure 5a,b are to compare prepared PGEs, and the summary of the data is presented in Table 2. Interception in the high-frequency region and x-axis in Nyquist plot (Real Z versus Imaginary Z) showed the bulk resistance $\left(R_{b}\right)$ of the PGEs. In general, Nyquist plots are separated into three regions, including semicircle at high frequencies, which is the interfacial charge transfer region, diffusion, and capacitive region. According to Figure 5a, the semicircles of Nyquist plots for the gel electrolytes were unnoticeable. This was because of the very small charge transfer resistance at the electrode-electrolyte interfaces [47,48].

The ionic conductivity, which can be extracted from the Nyquist plot, is a critical parameter in electrolyte properties. As bulk resistance increases, the conducting nature will decrease. Ionic conductivity (*σ*) of gels was calculated using Equation (1) and the resulted values were listed in Table 2:

(1) $$\sigma =\frac{L}{R_{b}A}\;$$

where L is the thickness of electrolyte and A is the contact area between graphite sheets and gels. Ionic conductivity values of the gel electrolytes were listed as 41.2, 58.8, 60, and 132 mS cm^−1^, respectively, for PGE, fCNT-PGE, GO-PGE, and fCNT/GO-PGE. The reason for the conductivity increment was that the Li^+^ with a small ionic radius diffused between GO layers and increased the final ionic conductivity. The charge transfer of fCNT/GO-PGE was three times higher than pure PGE electrolyte; it was also higher than fCNT-PGE and GO-PGE, for the distribution of carbon nanomaterials was more uniform in this gel electrolyte, facilitating the diffusion and migration of ions.

Bode plot is a frequency-based plot of EIS measurements, and the phase vs. frequency diagram includes three different areas: capacitive behavior at lower frequency, followed by diffusion and interfacial charge transfer [48]. The collected angle difference between the charging voltage and the current is presented as phase degree in Table 2. The negative angle degree is related to the leading current from voltage by 90 degrees [49]. Another parameter that was calculated from the bode plot was the average electrons lifetime (τ) by peak frequency (f_p_) as shown in Equation (2) [50].

(2) $$\tau =\frac{1}{2\;\pi f_{p}}$$

Since τ was as low as 0.001 mS for fCNT/GO-PGE, ions transferred faster, and this was in line with its higher ionic conductivity than other PGEs.

Electrochemical properties of gel electrolytes were further examined by cyclic voltammetry (CV) at 5, 10, 50, and 100 mV s^−1^ at room temperature. Symmetrical carbon cloth electrodes have been used as anode and cathode to establish cyclic voltammograms for a scan rate of 100 mV s^−1^ presented in Figure 5. The gel electrolytes incorporated with NCs showed better performance than PGE under all scan rates, which was due to the enhancement of ion transfer in the presence of fCNTs, GO and fCNT/GO in PGEs.

### 2.5. Galvanostatic Charge-Discharge Studies

**Figure 6:**
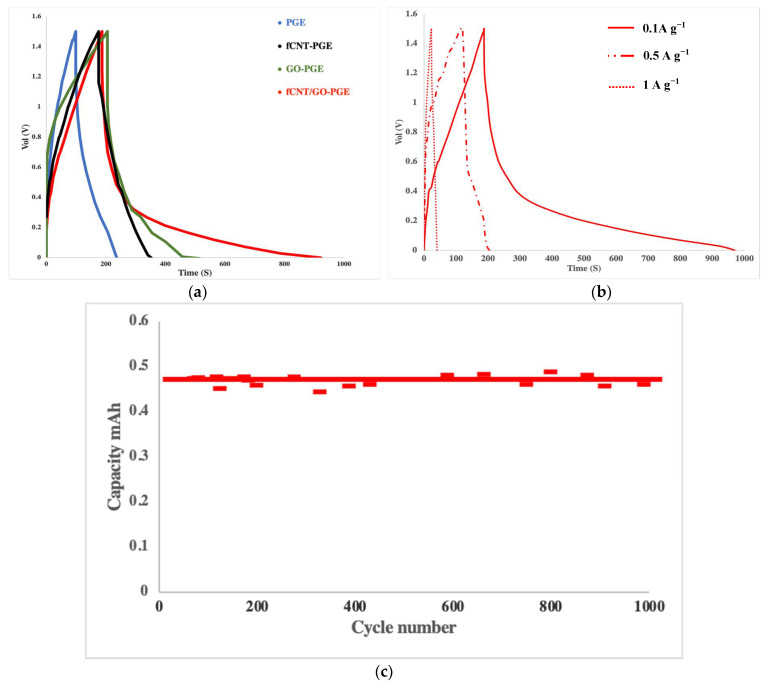
Electrochemical performance of GE based SCs, (i) PGE, (ii) fCNT-PGE, (iii) GO-PGE, (iv) fCNT/GO-PGE, (**a**) Galvanostatic Charge-Discharge curves under the current density of 0.1 A g^−1^, (**b**) Galvanostatic charge-discharge curves of fCNT/GO-PGE SC cell under the current density of 0.1, 0.5 and 1 A g^−1^, (**c**) Cycling performance of fCNT/GO-PGE SC cell at a current density of 1 A g^−1^.

The prepared electrodes and GEs were fabricated into supercapacitors and examined by galvanostatic charge-discharge measurements performed on an MTI Battery Analyzer. Supercapacitors were cycled between 0 and 1.5 V at the rate of 0.1 A g^−1^. Galvanostatic charge-discharge curves are presented in Figure 6. Capacitance was calculated from Equation (3) and listed in Table 2:

(3) $$C_{sp}:\;Specific\;capacitance\;\left(F\cdot g^{-1}\right)=\frac{I}{\frac{dV}{dt}\times m}\;$$

where *I* is discharge current (A cm^−2^), *dv/dt* is the slope of the discharge curve, and *m* is the mass of electrode material (g). Calculated capacitance values for fifth cycle were 39.5, 65.5, 77.6, and 83.3 F·g^−1^ for PGE, fCNT-PGE, GO-PGE, and fCNT/GO-PGE, respectively. The specific capacitances of SCs were improved by adding the NCs due to the increment of ionic conductivity in PGEs. The addition of the NCs in the PGE framework facilitated ion migration between the electrodes by generating nano pores and channels within the PGEs. fCNT/GO hybrid with a homogenous structure had less additive aggregation in composites and benefited from multiple parameters, demonstrating the best electrolyte properties. On the other hand, more gel-like properties of fCNT/GO-PGE provided better adhesion and fewer voids at the electrode-electrolyte interface. Charge-discharge curves of an fCNT/GO-PGE supercapacitor cycled at 1 A g^−1^, 0.5 A g^−1^, and 0.1 A g^−1^ are shown in Figure 6b. The performance retention was 98% after 1000 cycles, indicating high stability and reliability of the fCNT- and GO-doped polymer gel electrolyte (Figure 6c).

## 3. Materials and Methods

Acrylamide (AA), N, N′-methylene bisacrylamide (MBA), potassium persulfate, lithium sulfate, potassium permanganate, polyethylene oxide (PEO), carbon black, poly(3,4-ethylene dioxythiophene)-poly(styrene sulfonate) (PEDOT:PSS, 1.3 wt%), hydrochloric acid and sulfuric acid with the purity of 99+% were purchased from Sigma Aldrich. Carbon nanotubes (CNTs), graphene oxide (GO), and carbon cloth were purchased from Cheaptubes (Grafton, VT, USA), Graphenea (Cambridge, MA, USA), and the Fuel Cell Store (College Station, TX, USA), respectively.

### 3.1. Preparation of fCNT, fCNT/GO Composite

fCNTs and fCNT/GO composites were prepared by methods reported before [51,52]. In short, pristine CNTs were added to a mixture of concentrated HCl and H_2_SO_4_ (1:1 volume ratio) and reacted under 100 °C for 40 min. The sample was dried in a vacuum oven overnight after being filtered and triple washed to obtain fCNTs. Oxygen contents in the fCNTs were measured to be 18%. fCNT/GO composite was prepared by mixing 1:1 weight ratio of the two carbons and sonicating at room temperature for 3 h.

### 3.2. Synthesis of Polyacrylamide Gel Polymer Electrolytes

**Figure 7:**
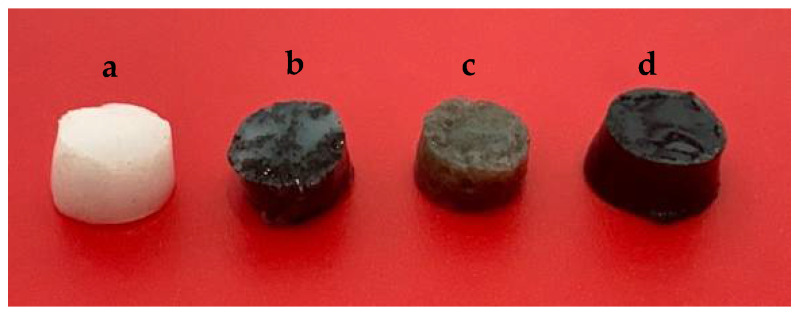
Images of (**a**) PGE, (**b**) fCNT-PGE, (**c**) GO-PGE, (**d**) fCNT/GO-PGE.

The plain gel PGE was synthesized by mixing a weighted amount of AA, MBA, and LiSO_4_ in MilliQ water and stirred at 80 °C for 1 h. This was followed by adding K_2_S_2_O_8_ aqueous solution and stirring for 1 min. The fluid was poured into a mold and gel was then generated within a few minutes. The amount of each component was fixed at 5.5% AA, 0.5% MBA, 18.5% LiSO_4_, 0.5% K_2_S_2_O_8_ and 75% water by weight. To incorporate carbon nanomaterials into the gel, one step was added to the procedure. In total, 1 mL of 0.1 mg/mL aqueous solutions of fCNTs, GO, fCNT/GO (0.6%) were sonicated with AA in water for 3 h prior to the first step of the synthesis. The rest of the process was kept the same as described before. Figure 7 presents image of prepared PGEs.

### 3.3. Electrode Preparation and Device Assembly

Manganese dioxide (MnO_2_) was synthesized as a base material for the electrodes. A total of 100 mL 0.25 M potassium permanganate solution and 9 mL concentrated hydrochloric acid was mixed and stirred overnight at room temperature. The procedure was followed by filtration and washing insoluble MnO_2_ using MiliQ water. The reaction is presented below:

2KMnO_4(aq)_ + 8HCl_(aq)_ → 2KCl_(aq)_+ 2MnO_2(s)_ + 4H_2_O+ 3Cl_2(g)_

The electrode composition was 85% MnO_2_, 5% PEO, and 10% carbon black. Total amount of 1 g dry powder was added into 2.4 g PEDOT:PSS solution, mixed and applied onto a conductive carbon cloth current collector. Total electrode materials were controlled at around 6 mg for further experiments.

**Figure 8:**
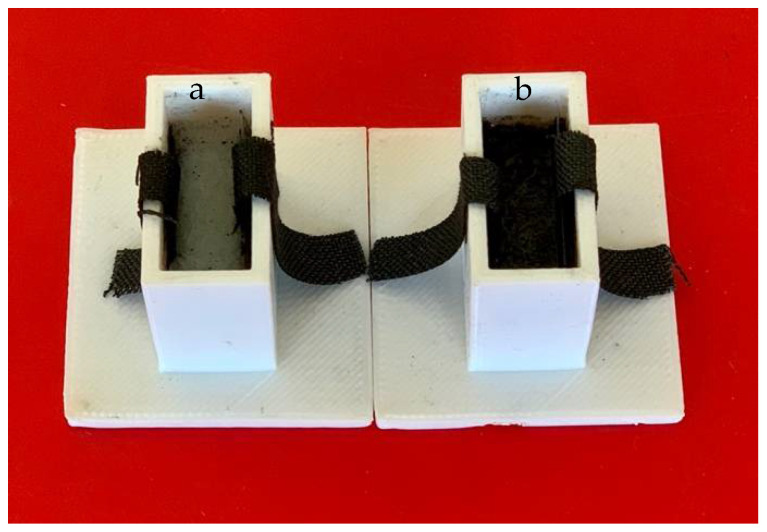
Supercapacitors made in 3D printed casings with different electrolytes inside: (**a**) PGE, (**b**) fCNT/GO-PGE.

Prepared electrodes were placed in the three-dimensional printed cases before gel electrolyte solutions were poured in for gelation processes. When the gels were totally formed, the supercapacitors were tested. 3D printed casing design and the assembled product are presented in Figure 8.

### 3.4. Characterization and Electrochemical Measurements

The morphologies of gel electrolytes were studied by JSM-7900F scanning electron microscope (SEM) from JEOL and Thermo Scientific DXRxi Raman imaging microscope. Thermal stability was investigated by Thermo Gravimetric Analyzer (TGA) and Differential Scanning Calorimeter (DSC) from Perkin Elmer. The rheological behaviors and mechanical properties of samples were studied by an oscillatory rheometer (Kinexus, Malvern Instruments, Malvern, UK). Electrochemical impedance spectroscopy (EIS) and cyclic voltammetry (CV) experiments were carried out on the Gamry instrument. Galvanostatic charge-discharge measurements were performed on an MTI Battery Analyzer.

## 4. Conclusions

In conclusion, PAM gel electrolytes doped with carbon nanomaterials were prepared, and their thermal, mechanical, and electrochemical properties were studied. PGE, fCNT-PGE, GO-PGE, and fCNT/GO-PGE electrolytes were compared. It was found that the incorporation of 0.6% carbon nanomaterials by weight altered the thermal and mechanical properties of PGE. The T_g_ of the samples increased from 93.7 for PGE to 130.8 °C for fCNT/GO-PGE. Using fCNTs along with GO in composite structures increased the synergetic effect and maintained the uniform distribution of integrated materials. Rheological properties of PGEs were studied, and elastic modulus was doubled in fCNT/GO-PGE compared to PGE owing to the hydrogen bonds formed between carboxyl groups in nano carbons and gel polymer chains.

Additionally, from EIS measurements, the ionic conductivity was doubled by adding fCNT/GO due to the formation of ion channels in the gel composition. The presence of fCNTs prevented aggregation of GO sheets and increased overall performance compared to fCNT-PGE and GO-PGE. By doping NCs into the GE, specific capacitance of SCs rose from 39.5 to 83.3 F·g^−1^. In general, doping of NCs, especially the combination of carboxylated nanotubes and graphene oxides, brought enhancement in thermal, mechanical, and electrochemical properties of PAM-based gel electrolytes. These gel electrolytes also hold great potential in flexible electrochemical device developments.

## Figures and Tables

**Table 1:** Thermal and mechanical properties of gels incorporated by carbon nanomaterials.

Gel Electrolytes	$T_{g}\left({}^{\circ}C\right)$	$\Delta H_{\;m}\left(\frac{J}{g}\right)$	Elastic Modulus (kPa)	Loss Factor (tan δ)
PGE	93.7	177.44	2.3	0.06
fCNT-PGE	118.5	169.44	1.5	0.05
GO-PGE	121.2	138.38	4.9	0.40
fCNT/GO-PGE	130.8	91.39	4.3	0.43

**Table 2:** Electrochemical properties of gels incorporated by carbon nanomaterials.

Gel Electrolytes	Bulk Resistance *R_b_* (Ω)	Ionic Conductivity σ (mS cm^−1^)	Phase (°)	Average Electrons LifeTime τ (mS)	Specific Capacitance (F·g^−1^)
PGE	16	41	−45	0.016	39.5
fCNT-PGE	11	59	−56	0.005	65.5
GO-PGE	10	64	−70	0.004	77.6
fCNT/GO-PGE	5	132	−83	0.001	83.3

## Data Availability

Data of this study are available from the authors upon request.

## Sample Availability

Samples of the composites are available from the authors upon request.
